# Current Concepts in Pediatric Philadelphia Chromosome-Positive Acute Lymphoblastic Leukemia

**DOI:** 10.3389/fonc.2014.00054

**Published:** 2014-03-25

**Authors:** Kathrin M. Bernt, Stephen P. Hunger

**Affiliations:** ^1^Department of Pediatrics, University of Colorado School of Medicine and Children’s Hospital Colorado, Aurora, CO, USA

**Keywords:** acute lymphoblastic leukemia, BCR–ABL1, tyrosine kinase inhibition, chemotherapy, hematopoietic stem-cell transplantation

## Abstract

The t(9;22)(q34;q11) or Philadelphia chromosome creates a BCR–ABL1 fusion gene encoding for a chimeric BCR–ABL1 protein. It is present in 3–4% of pediatric acute lymphoblastic leukemia (Ph^+^ ALL), and about 25% of adult ALL cases. Prior to the advent of tyrosine kinase inhibitors (TKI), Ph^+^ ALL was associated with a very poor prognosis despite the use of intensive chemotherapy and frequently hematopoietic stem-cell transplantation (HSCT) in first remission. The development of TKIs revolutionized the therapy of Ph^+^ ALL. Addition of the first generation ABL1 class TKI imatinib to intensive chemotherapy dramatically increased the survival for children with Ph^+^ ALL and established that many patients can be cured without HSCT. In parallel, the mechanistic understanding of Ph^+^ ALL expanded exponentially through careful mapping of pathways downstream of BCR–ABL1, the discovery of mutations in master regulators of B-cell development such as *IKZF1* (Ikaros), *PAX5*, and *early B-cell factor* (*EBF*), the recognition of the complex clonal architecture of Ph^+^ ALL, and the delineation of genomic, epigenetic, and signaling abnormalities contributing to relapse and resistance. Still, many important basic and clinical questions remain unanswered. Current clinical trials are testing second generation TKIs in patients with newly diagnosed Ph^+^ ALL. Neither the optimal duration of therapy nor the optimal chemotherapy backbone are currently defined. The role of HSCT in first remission and post-transplant TKI therapy also require further study. In addition, it will be crucial to continue to dig deeper into understanding Ph^+^ ALL at a mechanistic level, and translate findings into complementary targeted approaches. Expanding targeted therapies hold great promise to decrease toxicity and improve survival in this high-risk disease, which provides a paradigm for how targeted therapies can be incorporated into treatment of other high-risk leukemias.

Pharmacologic inhibition of the tyrosine kinase activity of BCR–ABL1 is the poster child for molecularly targeted cancer therapy. The first tyrosine kinase to be targeted, it is still the most effective “novel” therapeutic strategy to date, leading to remissions and possibly cures with single agents in chronic myeloid leukemia (CML) (1). Single-agent tyrosine kinase inhibition has not produced sustained responses in Ph^+^ ALL, but in combination with standard chemotherapy has revolutionized therapy and outcome for this patient population (2).

## The Biology of Philadelphia Chromosome-Positive ALL

### BCR–ABL1-induced leukemia

*BCR*–*ABL1* translocations are associated with two distinct clinical hematologic malignancies, CML and ALL. For CML, three discrete clinical stages have been defined: chronic phase, accelerated phase, and blast crisis. Genomic instability, the accumulation of additional cytogenetic (trisomy 8, isochromosome 17) and molecular (p53 pathway mutations, loss of p16^INK4A/ARF^) abnormalities, and BCR–ABL1-independent activation of downstream signaling pathways (LYN, AKT, STAT5) are all associated with – and likely contribute to – the progression to blast crisis (3). In about 30% of the cases, the predominant lineage in blast crisis is B-lymphoid rather than myeloid, speaking to the likely hematopoietic stem-cell origin of the disease. This presumed stem-cell origin may also explain the inability to achieve any durable remissions using conventional chemotherapy. Prior to the advent of tyrosine kinase inhibition, temporary disease stabilization was often achieved using hydroxyurea, low-dose cytarabine, and/or interferon, but the only curative approach was an allogeneic hematopoietic stem-cell transplantation (HSCT).

**Figure 1:**
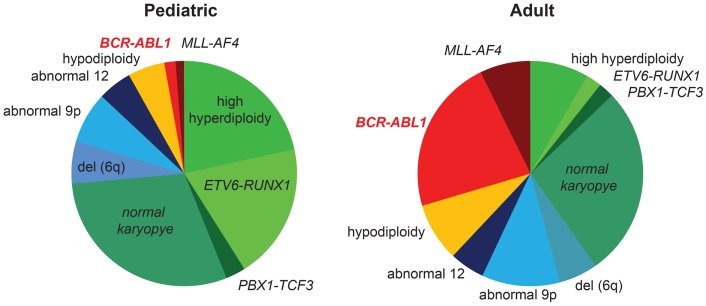
**Frequency of *BCR/ABL1* rearrangement in pediatric and adult ALL**. Cytogenetic abnormalities in pediatric (>1 year) and adult patients with ALL are shown (5). The majority of children <1 year of age carry a rearrangement of the *MLL*-gene and are not included in this graph. Favorable cytogenetic abnormalities are represented in green, neutral in blue, and unfavorable cytogenetics are represented in yellow/red. Favorable cytogenetics (high hyperdiploidy, *ETV6–RUNX1*) decrease, while the frequency of *BCR/ABL1* rearrangement increases with age. The higher percentage of unfavorable cytogenetics substantially contributes to inferior outcomes in adult versus pediatric ALL.

In addition to CML, *BCR–ABL1* translocations are found in a distinct subtype of ALL, called Ph^+^ ALL. The clinical presentation is indistinguishable from ALL with other cytogenetic abnormalities, and the diagnosis relies on the presence of the *BCR*–*ABL1* translocation (cytogenetics and FISH) and/or fusion transcript (PCR). Outcomes for Ph^+^ ALL were exceptionally poor when treated with chemotherapy, and HSCT in first remission was usually considered to be the best therapy (4). The frequency of *BCR–ABL1* rearrangement in ALL increases with age (Figure 1) (5) and has been reported as high as 50% in the elderly (6). A greater percentage of patients with adverse cytogenetics contributes substantially to the overall worse outcome in adult compared to pediatric ALL (Figure 1).

The first indication that the BCR–ABL1 fusion protein is indeed the crucial driver of CML came from mouse studies showing that expression of BCR–ABL1 in the bone marrow causes a CML-like disease (7–9). Studies that utilized a mutant BCR–ABL1 protein with an inactive tyrosine kinase domain defined the tyrosine kinase activity of ABL1 as absolutely required for transformation (10). This suggested that targeted inhibition of the ABL kinase domain might be an effective therapeutic strategy in BCR–ABL1-driven hematologic malignancies. The pioneering work of Brian Druker spearheaded the clinical development of the first tyrosine kinase inhibitor (TKI), Imatinib (11–13). Imatinib gained FDA approval in 2000 and revolutionized CML therapy, converting a near universally fatal disease requiring HSCT into a chronic condition controlled with monotherapy of a targeted agent (14). In the years since the initial success of imatinib, second [nilotinib, dasatinib, bosutinib (15–17)] and third [ponatinib (18)] generation ABL1 class TKIs have been developed, which are active against multiple imatinib-resistant BCR–ABL1 mutants.

Early studies using imatinib as monotherapy in Ph^+^ ALL were disappointing, with initial responses rapidly progressing to TKI-resistant disease. However, the integration of TKIs into a high-risk ALL chemotherapy backbone fundamentally changed our approach to Ph^+^ ALL as well. Overall survival (OS) using this strategy more than doubled compared to chemotherapy-only treated historic controls (2), and HSCT is no longer universally recommended for Ph^+^ ALL. Despite these advances, the survival of Ph^+^ ALL still lags behind most other cytogenetic subgroups in pediatric ALL. A better understanding of the biology of Ph^+^ ALL may help to refine therapy and develop rational combinations of targeted agents that will further improve outcomes for patients with this disease.

### The Philadelphia chromosome and BCR–ABL1 fusion

**Figure 2:**
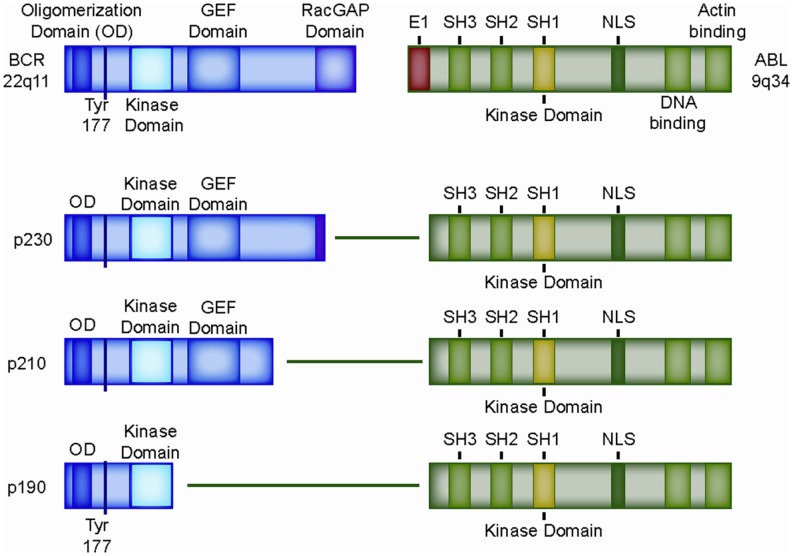
**Structure of the most common BCR–ABL1 fusion genes**. Domain structure of wild type BCR and wild type ABL1 protein, as well as retained domains in the three most common BCR–ABL1 variants, p230, p210, and p190. OD: oligomerization domain (coiled-coil domain) mediating oligomerization, Tyr177: tyrosine 177, which, when phosphorylated, serves as a docking site for the adaptor protein GRB-2; SH2-domain: SRC homology 2 (binding to phosphorylated tyrosine residues, including BCR exon 1), SH3-domain: SRC homology 2 (binding to proline rich peptides). SH1-domain: SRC homology 1 (ABL1 catalytic domain); GEF-domain: guanine nucleotide exchange factors (G-protein signaling); E1: exon 1 of ABL1, contains the inhibitory N-terminal “cap” that binds the catalytic domain (SH1) of ABL1 and prevents autophosphorylation; NLS: nuclear localization signal.

Ph^+^ ALL derives its name from the presence of the Philadelphia (Ph) chromosome, named after the city where it was first described in the leukemia cells of a CML patient by Nowell and Hungerford in 1960 (19). In 1973, Janet Rowley reported that the Philadelphia Chromosome was the der(22) product of the reciprocal t(9;22)(q34;q11.2) translocation (20). The *BCR–ABL1* fusion gene is generated by joining almost the entire coding region of the *ABL1* tyrosine kinase gene (Abelson murine leukemia virus homolog, exons 2–11, chromosome 9) to the breakpoint cluster region (*BCR*) gene on chromosome 22 (Figure 2) (21). There are two main regions where breakpoints cluster within the *BCR* gene. The “CML” breakpoint region lies between exons 12 and 16 in a region called the major breakpoint cluster region (M-BCR). Translocations involving the M-BCR produce the larger p210 BCR–ABL1 protein, which derives its name from its molecular size of 210 kDa. Translocations that occur within the minor “ALL” BCR (m-BCR) yield a smaller p190 gene product that retains only the first exon of *BCR*. A rare p230 fusion protein (with a “micro”-BCR breakpoint between exons 19 and 20) has also been described. Both p210 and p190 transform primary human and murine bone marrow cells (8, 9, 22). Of the two, the “ALL-type” p190 is the stronger transforming agent (7, 23). About 90% of pediatric Ph^+^ ALL patients have the classic ALL-type p190 translocation (24) with some variability reported in the literature as CML in B-lymphoid blast crisis can sometimes be hard to distinguish from Ph^+^ ALL.

Wild type ABL1 is a ubiquitously expressed but tightly regulated non-receptor tyrosine kinase that is present throughout hematopoietic development, with declining levels during myeloid maturation. It is predominantly located in the cytoplasm in hematopoietic cells, but can shuttle to the nucleus. In the cytoplasm, ABL1 is found mostly bound to actin, and functions include signaling and modulation of the cytoskeleton. Nuclear ABL1 has been implicated in cell cycle control. The N-terminus of ABL1 negatively regulates ABL1 kinase activity, allowing for tight titration of ABL1 kinase activity under physiologic conditions. Loss of the N-terminus as a result of the *BCR–ABL1* translocation results in high constitutive kinase activity. Thus loss of this important regulatory domain is a major contributor to ABL1-mediated leukemogenesis (25) [reviewed in Ref. (26)].

The fusion partner of *ABL1, BCR*, is a complex locus that is transcribed into two major proteins, both with multiple functional domains implicated in a variety of fundamental biological processes. These include G-protein signaling pathways, cytoskeletal organization, growth, and development. The only exon of BCR that is consistently retained in all fusions is exon 1, which encodes a coiled-coil domain facilitating dimerization and autophosphorylation (amino acids 1–63) (27, 28), a docking site for the adaptor protein GRB-2 (phosphorylated tyrosine 177) (28, 29), and a tyrosine kinase domain (amino acids 298–413) (30). The exact role of the BCR-tyrosine kinase domain is unclear, and in a murine model of CML utilizing retroviral introduction of p210_BCR–ABL1_ deletion mutants into bone marrow cells, it appeared to be dispensable. On the other hand, the consistent inclusion of the BCR-tyrosine kinase domain in human BCR–ABL1-driven malignancies suggests that it may play a functional role in leukemogenesis (30–32).

### Downstream pathways activated by BCR–ABL1 fusion proteins

**Figure 3:**
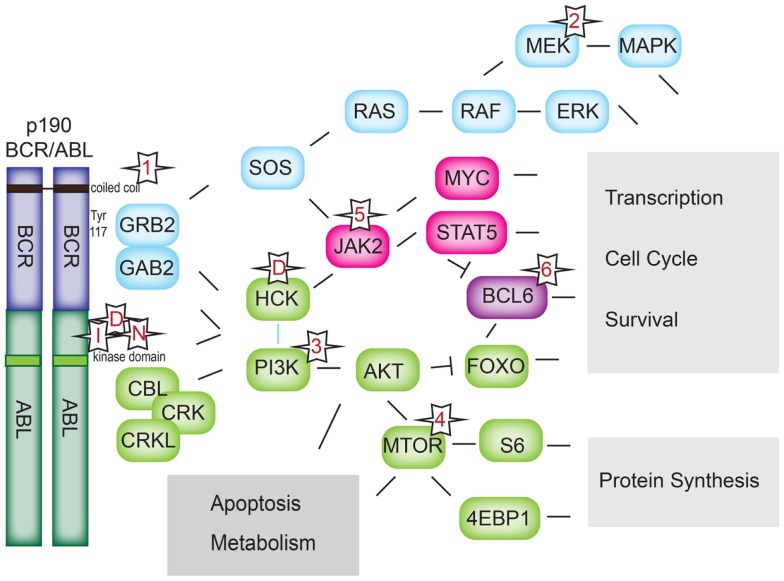
**BCR–ABL1 signaling pathways**. Downstream signaling pathways activated by BCR–ABL1. Numerical references 1–6 denote classes of inhibitors in Table 1. I: imatinib; D: dasatinib, N: nilotinib.

The molecular consequence of all BCR–ABL1 fusion proteins is a hyperactive ABL1 kinase domain and aberrant phosphorylation of a variety of targets. Activation results from lack of autoinhibition due to loss of the N-terminal regulatory domain of ABL1, and homodimerization and autophosphorylation of the fusion protein (27). The importance of the homodimerization and autophosphorylation for BCR–ABL1 signaling is underscored by promising *in vitro* results of peptides and small-molecule inhibitors that cause allosteric inhibition of BCR–ABL1 (33–36). BCR–ABL1 kinase activity leads to direct and indirect activation of multiple pathways (37), including PI3K (38), AKT (39–41), MTOR (42, 43), RAS (39, 44), EGFR, MAP-kinase (40, 43, 45), JNK/SAPK (43), JAK1–3 (46), the SRC-family kinases LYN, HCK, and FGR (47), PTPN11, NF-kB, phospholipase C, and, as a common downstream effector of many of these pathways, STAT5 (Figure 3) (46, 48–50). Most of these pathways have been worked out in CML, but the relevant binding sites or kinase domains are preserved in the p190 fusion protein. Activation of JAK1–3 (50) and STAT1, 3, 5, and 6 (48, 50) has been experimentally confirmed for p190. Work in CML suggests that JAK1–3 activation is mediated through the interaction of BCR–ABL1 with cytokine receptors rather than direct phosphorylation (51). On the other hand, JAK2 appears to directly phosphorylate BCR–ABL1 at the critical tyrosine-177 residue and increase BCR–ABL1 protein stability, thus enhancing BCR–ABL1 signaling (52). Another important downstream pathway that has been confirmed specifically in Ph^+^ ALL is the PI3K–AKT–MTOR pathway. Deletion of PI3K inhibited leukemogenesis in a murine model of p190 Ph^+^ ALL. A dual PI3K/MTOR inhibitor was effective on Ph^+^ ALL patient samples (53) and showed synergy with Imatinib (54). The activation of AKT and MTOR signaling also plays a critical role in steroid resistance in ALL (55, 56), and multiple agents targeting the PI3K/AKT/MTOR axis are currently in clinical trials for pediatric ALL [reviewed in Ref. (57)].

**Table 1:** **BCR–ABL1 downstream and parallel pathways as drug targets**.

Gene	Evidence for role in BCR–ABL1-driven leukemia	Drug
BCR–ABL1	Allosteric inhibition of homodimerization and autophosphorylation using GNF2 and GNF5 has activity in retroviral BCR–ABL1 mouse models (incl. T315I BCR–ABL1), particularly in combination with TKIs (35, 36)	*GNF2, GNF5*^1^
GRB-2	Impaired binding to p210 BCR Tyr117Phe results in complete loss of leukemogenicity in retroviral BCR–ABL1 mouse model (29, 248), knockdown decreases proliferation of BCR–ABL1 transduced human CD34 cells (45). Validated targets: RAS (39), MAPK, AKT (39)	
GAB-2	Ligand mimetic inhibitory peptide induces apoptosis in K562 (CML) cells (62)	
RAS	Activated in human CD34+ cells after transduction with p210 BCR–ABL1, dependent on Tyr177 (39)	
MAPK/MEK	Increased in response to imatinib (p210 human CD34+ model), MEK-inhibitor reduces proliferation, synergy with imatinib (40). No Mek inhibition currently in trials for leukemia, phase I study of sorafenib in relapsed leukemia completed (Raf-kinase inhibitor, also inh. VEGFR2/PDGFRβ, NCT00131989)	Trametinib^2^, selumetinib MEK162, PD325901
PI3K/AKT	Activated in human CD34+ cells after transduction with BCR–ABL1, dependent on Tyr177, PI3K inhibitor effective in p210 human CD34+ *in vivo* model (39, 40), Ph^+^ ALL mouse model, and Ph^+^ ALL patient cells (41). Akt activation plays a role in steroid resistance (55). Pi3K inhibitors in trials for relapsed leukemia: BKM120, BEZ235, AMG319, idelasilib	Perifosine, IPI145^3^, idelalisib, PX866, BAY80-6946, SF1126, many more
MTOR	PI3K–AKT–MTOR pathway activated by BCR–ABL1. Rapamycin (sirolimus) and dual PI3K/MTOR inhibitor effective *in vitro* on Ph^+^ ALL patient samples and synergy with TKI (54, 171). MTOR has also been implicated in mediating steroid resistance (56). Multiple agents in clinical trials in relapsed/refractory ALL, including TORC1/2 inhibitors (AP23573) for relapsed leukemia	Sirolimus^4^, everolimus temsirolimus TORC1/2 inhibitors
JAK1/2/3	JAK1, 2, 3 and STAT1, 3, 5, 6 are activated in response to BCR–ABL1 signaling in p190 transduced cells, JAK2 also directly phosphorylates BCR–ABL1. JAK2 knockdown and dual JAK2/BCR–ABL1 inhibition impairs CML cell lines *in vitro* and in xenografts, and CML cells *in vitro* (52, 249)	Ruxolitinib (Jak1/2)^5^ *ONO44580* (Jak2–BCR–ABL1)
FRG	Src-family, required in p210 lymphoid leukemia mouse model, significant redundancy with other family members (47)	Dasatinib^D^ (Src-family + BCR–ABL1)
Hck	Src-family, required in p210 lymphoid leukemia mouse model, significant redundancy with other family members (47)	Dasatinib^D^ (Src-family + BCR–ABL1)
Lyn	Src-family, required in p210 lymphoid leukemia mouse model, significant redundancy with other family members (47)	Dasatinib^D^ (Src-family + BCR–ABL1)
ERBB	Overexpressed in Ph^+^ ALL (56 versus 4.8% of Ph^−^ ALL). Lapatinib synergistic with imatinib and nilotinib (but not dasatinib) on Ph^+^ ALL cell lines (63)	Lapatinib (ERBB/HER2/NEU)
BCL6	Upregulated in response to TKI in Ph^+^ ALL; deletion, dominant negative, or pharmacologic inhibition synergizes with imatinib and nilotinib in mice (188)	*RI-BPI, C79-6^6^*(188, 250, 251)
MDR1	Promoter methylation inversely associated with presence of Ph^+^ (146)	
LRP	Expression increased compared to normal bone marrow (179)	

*Brief summary of pathways implicated in leukemogenesis and resistance in BCR–ABL1-positive leukemia, as well as potential agents targeting the respective pathways. Superscript numbers and alphabets denote inhibitors of pathways in Figure 3. TORC1/2: MTOR activates two major downstream complexes, TORC1 and TORC2, with distinct biological functions. Sirolimus (rapamycin), temsirolimus, and everolimus inhibit TORC1. Newer generation MTOR inhibitors that target both TORC1 and TORC2 are also currently in clinical trials. *Italics*: *compounds in preclinical development**.

Of particular importance appears to be the adaptor proteins GRB-2 (58–60) and GAB-2 (60), which interact with, and participate in the activation of nearly all of the signaling pathways cited above. GRB-2 has been shown to bind phosphorylated tyrosine-177 (Figures 2 and 3). The importance of this interaction is demonstrated by the impaired *in vivo* leukemogenesis in murine models of p210_BCR–ABL1_ constructs with an engineered inactivating mutation of tyrosine-177 (29, 45, 61). Peptide-inhibition of the SH3 domain of the adaptor protein GRB-2 reduced growth and induced apoptosis in the *BCR*–*ABL1*-positive K562 cell line (7). Similarly, genetic inactivation of GAB-2 impairs p210_BCR–ABL1_-mediated transformation in mice (62).

Recently, overexpression of the epidermal growth factor ERBB was found to be specifically elevated in Ph^+^ ALL (56 versus 4.8% of Ph^−^ ALL) (63). The molecular details of how this pathway intersects with BCR–ABL1 signaling requires further study; preliminarily, p70S6 kinase (MTOR target) has been implicated. From a translational perspective, the ERBB/HER2/NEU inhibitor lapatinib was synergistic with imatinib and nilotinib (but not dasatinib) on Ph^+^ ALL cell lines.

The reported activation of the SCR-family kinases LYN, HCK, and FGR by BCR–ABL1 has important implications. BCR–ABL1 has been shown to interact with and activate SRC-family kinases, and inhibition of SRK-family kinases decreased growth and survival of *BCR*–*ABL1-*positive myeloid cell lines *in vitro* (64–71). In addition to being activated by BCR–ABL1, the Src-family kinases Lyn (72) and Hck (73) have been reported to in turn phosphorylate BCR–ABL1 at several sites, including the critical residue for the interaction with the adaptor proteins Grb-2 and Gab-2 (Tyrosine-177). Expression of p210_BCR–ABL1_ in murine lymphoid progenitors negative for all three kinases (*Lyn^−/−^ Hck^−/−^ Fgr^−/−^*) near completely prevented leukemogenesis in a mouse model of Ph^+^ lymphoid leukemia (47). There was considerable redundancy between Lyn, Hck, and Fgr in this model, and genetic inactivation of at least two kinases was required to protect mice from leukemia. Somewhat surprisingly, given that CML cell lines responded to Src inhibition *in vitro* (67–71), *Lyn, Hck*, and *Fgr* were not required to induce CML *in vivo* (47). A small-molecule inhibitor of SRC-family kinases improved the survival of mice with lymphoid but not myeloid leukemia. Lack of inhibition of Src-family kinases by imatinib, and dual inhibition of Src-kinases and BCR–ABL1 with dasatinib were proposed to underlie the improved efficacy of dasatinib in the p210 lymphoid leukemia model (74). Under normal physiologic conditions, *Lyn^−/−^ Hck^−/−^ FGR^−/−^* mice display defects in B-cell maturation and autoimmune features suggesting a specific role for these kinases in B-cell development, but the early B-cell compartments appear to be preserved (47). Dependency of Src-family kinases may thus be a specific feature of Ph^+^ ALL. This is highly relevant from a clinical–translational standpoint as it provides a compelling rationale to investigate the dual BCR–ABL1–SRC-family kinase inhibitor dasatinib in Ph^+^ ALL, and suggests that this agent may be more effective in Ph^+^ ALL than imatinib or nilotinib.

Many of these downstream pathways – particularly the JAK–STAT pathway, are also targeted by several newly described leukemogenic fusion proteins that induce a disease similar to Ph^+^ ALL, but without a *BCR*–*ABL1* rearrangement. These “Ph-like” leukemias share with Ph^+^ ALL a transcriptional signature indicative of kinase activation, co-occurring mutations in the B-cell transcription factor *IKZF1*, and a poor outcome. Initially identified based solely on transcription profiling, improved molecular techniques have allowed identification of a tyrosine kinase mutation in many of these patients. These include rearrangements of *JAK2, ABL1, PDGFRB, CRLF2*, and *EPOR*, deletion of *SH2B3* encoding the JAK2-negative regulator LNK, and activating mutations of *FLT3* and the IL7 receptor (*IL7R*) (75, 76). The presence of these mutations opens the door for potential therapeutic impact using targeted inhibitors (77). This fascinating subgroup of ALL is the topic of a dedicated review in this issue.

### Differences between p210 and p190 – lessons from mouse models

As mentioned earlier, p190_BCR–ABL1_ has stronger transforming activity than p210_BCR–ABL1_, both in fibroblast transforming assays (78) and in mouse models (7, 79). One possible reason may be a higher specific kinase activity and possibly broader substrate range of the p190_BCR–ABL1_ fusion protein (78). In transgenic animals, p190_BCR–ABL1_-induced exclusively B-lymphoid leukemia with a short latency, while p210_BCR–ABL1_ led to development of both lymphoid and myeloid leukemias with a longer latency (79). When introduced into stem cell and progenitor enriched mouse bone marrow, both p210_BCR–ABL1_ and p190_BCR–ABL1_ cause a myeloproliferative disease with expansion of granulocytic, myelomonocytic, and lymphoid compartments; however, the disease induced by p190_BCR–ABL1_ has a significantly shorter latency (7). p190_BCR–ABL1_ induces stronger STAT1 and STAT5 phosphorylation in Baf3 cells than p210_BCR–ABL1_ (48), and also induces phosphorylation of STAT6 (50).

### Co-occurring genetic abnormalities

Next generation sequencing studies have revealed that many leukemia genomes are remarkably stable – particularly when compared to epithelial cancers. Nevertheless, Ph^+^ ALL cells have been shown to carry several recurrent mutations that commonly co-occur with *BCR*–*ABL1* fusions and contribute to leukemogenesis. The most frequent co-occurring genetic abnormalities are deletions of the lymphoid-specific transcriptional regulators *IKAROS* (*IKZF1*), *PAX5 (paired box 5)*, and *EBF1 (early B-cell factor 1)*. Deletions involving *CDKN2A/B* are also common. In addition, one of the first examples of “convergent clonal evolution” within the same leukemia was described in Ph^+^ ALL: one patient’s leukemia contained two cytogenetically distinct subclones that independently acquired a duplication of 8q, corroborating the crucial role of co-occurring mutations (80). Interestingly, GWAS studies have identified genetic polymorphisms of *IKZF1* (81–87), *PAX5* (88), and *CDKN2A/B* (81, 89–91) as susceptibility loci that mediate a genetic predisposition to childhood ALL. However, subgroup analysis, when performed, revealed no specific association with Ph^+^ ALL. This may in part be due to a low number of Ph^+^ ALL patients in these studies, and a targeted evaluation of *IKZF1, PAX5*, and *CDKN2A/B* susceptibility alleles specifically in Ph^+^ ALL patients may be warranted.

### IKZF1 deletions and point mutations in Ph^+^ ALL

A review of *BCR*–*ABL1* in ALL requires discussion of its most frequent partner in crime, *IKZF1* (86). *BCR*–*ABL1* and *IKZF1* mutations are strongly linked: about 70–80% of Ph^+^ ALLs have somatic mutations in *IKZF1* (about 90% deletions and 10% point mutations), which is much higher than the rate of *IKZF1* mutations in Ph^−^ ALL (92–94). There are three functional types of *IKZF1* mutations: haploinsufficiency or near haploinsufficiency (due to monoallelic null mutations such as inactivating point mutations, premature stop codons, and deletions, 55%), complete absence of Ikaros due to bi-allelic deletions (12%) (92, 93, 95), and alterations that create a dominant-negative (DN) form of Ikaros, IK6 (33% of all *IKZF1* mutations). The IK6 Ikaros mutant is produced by an in-frame deletion of exons 4–7, which deletes the DNA-binding domain and leads to cytosolic accumulation of the mutant protein (92, 96, 97). The resulting hematopoietic phenotype in a mouse model mimicking this mutation (a smaller deletion that phenocopies the loss of the DNA-binding and nuclear export) is more severe that monoallelic null mutations, as Ik6 associates with the wild type Ikaros and probably traps it in the cytoplasm together with other complex members such as Helios, Aiolos, and Eos (98). The mutations associated with a more profound reduction in Ikaros function (bi-allelic deletion and Ik6) are particularly common in Ph^+^ ALL (92, 93, 95–97). This underscores the remarkably tight link between Ikaros and Ph^+^ ALL. In the closely related “Ph-like” ALL subset characterized by a gene-expression profile highly similar to that of Ph^+^ ALL, but without *BCR*–*ABL1* fusion, *IKZF1* mutations are also common. However, the majority of mutations result in a less severe reduction of Ikaros function [i.e., haploinsufficiency in 55–70% of all *IKZF1* mutations in non-*BCR*–*ABL1* ALL (95, 99)]. Twin studies and tracking of subclonal populations suggests that *BCR*–*ABL1* fusion is the first hit, and *IKZF1* mutations occur later during leukemogenesis (95, 100, 101). There are also reported cases of “convergent” evolution of *IKZF1* mutations, with different subclones within the same patient carrying different *IKZF1* mutations, underscoring the importance of this locus for Ph^+^ ALL (72, 80, 101). Much work has been dedicated to understanding the molecular mechanism of loss of Ikaros alone and in the context of Ph^+^ ALL. Complete loss or expression of DN Ikaros in normal hematopoiesis causes a mild (*Ikzf1^−/−^*) to severe (DN) reduction in the number of hematopoietic stem cells (HSC), complete loss of the B-cell and dendritic cell compartments, a skewing toward the T-lymphoid lineage (98, 102–108), and ultimately T-cell malignancies in mice (109). Despite this, *IKZF1* mutations are much more common in B-lymphoid than in T-lymphoid malignancies (110). On the surface, the combination of *BCR*–*ABL1* fusion and loss of *IKZF1* neatly fits the paradigm proposed by Gilliand for acute myeloid leukemia (AML), which hypothesized that leukemia development requires a combination of class I (signal transduction pathway mutation leading to uncontrolled growth, such as *FLT3*, or *RAS*), and class II mutations (aberrant transcription factors resulting in differentiation block, such as *PML–RAR, AML–ETO, MLL*-translocations, or point mutations in C/EBPα) (111). According to this model, *BCR*–*ABL1* is the class I mutation and *IKZF1*, the class II mutation. A possible reason for the frequent occurrence of at least the Ik6 mutation in B-cell precursor ALL could lie in the fact that the exons 4 and 7 are flanked by genomic regions that can function as off-target sites for recombination activated gene (RAG) proteins, which mediate VDJ recombination in this cell population (93, 112). Whether the particularly common co-occurrence of BCR–ABL1 and Ik6 is solely a function of the developmental stage of the cell of origin, or whether the presence of the *BCR*–*ABL1* translocation predisposes to aberrant RAG activity is not known. In addition, if and how BCR–ABL1 and mutant Ikaros cooperate on a molecular level is still not fully understood. The normal function of Ikaros suggests that one of its main contributions to B-cell leukemogenesis is a differentiation block in the B-lymphoid lineage at the pro- to pre-B-cell transition. While complete loss of Ikaros results in a complete absence of the entire B-cell compartment, a severely reduced expression of Ikaros allows development up to the Pro-B-cell stage but not beyond (113). However, additional mechanisms likely play a role. Ikaros has been shown to downregulate Myc, thus loss of Ikaros may result in increased Myc activity and increased proliferation (114). Gene-expression profiling suggests that *IKAROS* mutated B-ALL has a more prominent “stem-cell signature” (99, 115), and a larger leukemia initiating cell pool [LIC, defined by CD34 expression rather than functionally (96)], suggesting that some of the functions of Ikaros in silencing stem-cell programs in HSCs may play a role (105, 116). Finally, it has been suggested that loss of Ikaros may either synergize with or enhance Jak–Stat signaling. This hypothesis is mostly based on the circumstantial evidence that the other main subtype of ALL with frequent *IKZF1* mutations are the Ph-like leukemias. Ph-like leukemias share a transcriptional profile with Ph^+^ ALL and frequently carry mutations that, like BCR–ABL1, activate Stat5 (75, 117). Modulation of this pathway could both provide a competitive advantage at a subclonal level of *IKZF1^−^* clones, and provide an escape pathway for BCR–ABL1 inhibition. Loss of Ikaros predicts a poor prognosis even within Ph^+^ ALL (94, 99).

### PAX5 mutations in Ph^+^ ALL

**Figure 4:**
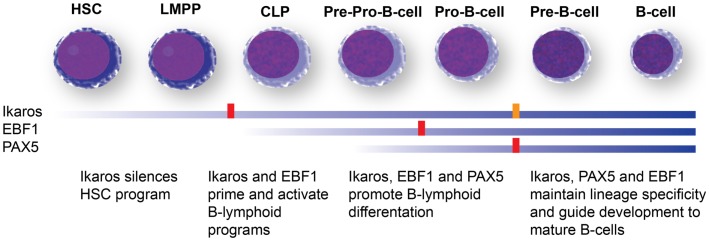
**B-cell development and transcription factors mutated in Ph^+^ ALL**. Differentiation stage-dependent expression (blue bars) and function of the three major B-cell developmental regulators mutated in Ph^+^ ALL, Ikaros, Pax5, and EBF1 (106, 107). Ikaros expression is detected early in hematopoietic development and appears to have a role in shutting down stem-cell programs and nudging cells toward lymphoid development. Ikaros expression is maintained through B-cell development. Complete loss of Ikaros in murine models leads to a differentiation block at the LMPP stage and complete absence of the entire B-cell lineage (red block). A severe reduction allows the development of B-cell progenitors, but maturation is blocked at the Pro-B stage (orange block). EBF1 is turned on in common lymphoid progenitors (CLPs) and controls lineage specification to the B-cell lineage. Loss of EBF1 in murine models leads to a differentiation block at the Pro-B stage (red block). Pax5 is turned on the latest and maintains lineage commitment. Loss of Pax5 causes a differentiation block at the Pro-B-cell stage. Neither Pax5 nor EBF1 appear to have a role in silencing hematopoietic stem-cell programs, which may explain why *IKZF1* mutations are associated with a poor prognosis, while *PAX5* and *EBF1* mutations do not predict adverse outcomes. HSC: hematopoietic stem cells; LMPP: lymphoid-primed multipotent progenitors; CLP: common lymphoid progenitors.

Recurrent mutations of *PAX5* occur in about one-third of B-ALL cases (99, 118, 119), and in up to 50% of Ph^+^ ALL (92, 120, 121). PAX 5 is a transcription factor that is expressed specifically during B-cell development, and controls lineage identity and commitment (107, 122). Like loss of Ikaros, loss of Pax5 leads to a differentiation block at the pro- to pre-B-cell stage (122–124). Loss of Pax5 also allows trans-differentiation of already lineage-committed pro-B cells into other lineages, confers a certain degree of self-renewal onto this population (125, 126), and can cause B-cell lymphomas (127). Unlike Ikaros, however, the physiologic expression of PAX5 is limited to B-cell precursor stages, and its loss is not associated with an adverse prognosis (99, 120, 121). It has been speculated that a lack of an effect of PAX5 on hematopoietic stem-cell transcriptional programs may be responsible for the different prognostic implications of *PAX5* and *IKZF1* mutations (120, 128). In addition to driving B-lymphoid development, Ikaros has been reported to repress hematopoietic stem-cell specific gene-expression programs during early lineage specification, a function not shared with the other two major regulators of B-cell development that are found mutated in Ph^+^ ALL, PAX5, and EBF1 (Figure 4) (105, 116, 128).

### EBF1 mutations in Ph^+^ ALL

Early B-cell factor 1 is the predominant transcription factor mediating B-cell lineage commitment (107). It has been shown to co-regulate target genes with PAX5. In mouse models, complete loss of *Ebf1* leads to a differentiation block at the pre–pro-B-cell stage (129). In contrast to *Ikzf1* and *Pax5, Ebf1^−/−^* mice do not develop spontaneous hematologic malignancies (129). However, combining loss of one allele of *Ebf1* or *Pax5* with a constitutively active *Stat5* allele (the downstream effector in both Ph^+^ and Ph-like ALL) results in B-cell precursor leukemia in all animals (130). *EBF1* mutations occur in about 14% of Ph^+^ ALL.

### CDKN2A/B in Ph^+^ ALL

The *CDKN2A/B* locus is frequently altered in ALL. The products of the *CDKN2A* and *CDKN2B* genes, p16^INK4A^ and p15^INK4B^, are inhibitors of cyclin-dependent kinases. In addition, transcription of an alternate reading frame of the CDKN2A locus produces p14^ARF^, which antagonizes the p53 ubiquitin ligase, HDM2. Silencing of the *CDKN2A/B* locus in HSC has been implicated in HSC self-renewal (131–134). The distribution of *CDKN2A/B* alterations within cytogenetic subgroups is non-random. *CDKN2A/B* is rarely deleted in ALL with translocations of *E2A* [*E2A–PBX1* in t(1;19)(q23;p13) and *E2A*–*HLF* in t(17;19)(q21–22;p13) ALL] (135). In contrast, increased rates of *CDKN2A/B* deletions are found in Ph^+^ ALL with a reported frequency of ~50% (80, 92, 136) compared to around 30% in non-Ph^+^ B-ALL (137, 138). *CDKN2A/B* deletions are rare in CML in chronic phase but frequently associated with the transformation to lymphoid blast crisis, suggesting a specific role in B-lymphoid leukemia (139). Similar to *IKZF1*, the mechanism of deletion of p16 in lymphoid malignancies may involve RAG-mediated recombination (140). Experimental overexpression of BCR–ABL1 induces expression of Arf, which, if unopposed, leads to apoptosis (141). Introduction of p190_BCR–ABL1_ into *Arf* -null murine bone marrow decreases the latency and increases resistance to imatinib in the lymphoid malignancy that develops in recipient mice. In most clinical studies that have assessed the prognostic significance of *CDKN2A/B* loss of function in ALL, *CDKN2A/B* deletion or hypermethylation do not appear to be associated with changes in outcome for pediatric ALL, while silencing or inactivation of the locus predicts a worse outcome in adults (99, 136, 138, 142–146). In a recent study mapping clonal evolution in adult patient-derived Ph^+^ ALL cells grown in immunodeficient mice, the loss of *CDKN2A/B* was associated with increased competitive advantage on a subclonal level, more aggressive growth in xenografts, a higher leukemia initiating frequency, and a trend toward inferior outcome in patients (80). In both children and adults, deletions as well as epigenetic silencing through promoter hypermethylation are found at increased frequencies in relapsed specimens as opposed to those from initial diagnosis, suggesting a role in mediating relapse and resistance to therapy (147–150).

### Epigenetic abnormalities in Ph^+^ ALL

In addition to genetic abnormalities, Ph^+^ ALL has a characteristic DNA methylation profile. Ph^+^ ALL can be distinguished from other subtypes of ALL by hierarchical clustering of DNA methylation profiles. A recent study that quantified differentially methylated regions (DMRs) in all major ALL subtypes (compared to B-cell precursors, i.e., using a developmentally matched control) revealed about 350 DMRs in Ph^+^ ALL samples (151). This was remarkably different from only about 50 DMRs identified in *CRLF2*-rearranged ALL samples, many of which were “Ph-like” ALL samples that share a major transcriptional program with Ph^+^ ALL but are negative for the *BCR*–*ABL1* translocation, and instead commonly have activating Jak1/2 mutations. Whether differential methylation is a consequence of the BCR–ABL1 fusions or co-occurring genetic abnormalities, and whether it plays a role in malignant transformation, resistance or relapse is unknown. However, an active role for DNA methylation in malignant transformation (rather than a mere reflection of the transcriptional landscape) is supported by the dependence of several experimental tumor models on functional DNA methyltransferase Dnmt1 (152, 153), including MLL–AF9 and Myc–Bcl2-driven leukemia (154, 155). Reactivation of silenced CDKN2A/B by demethylating agents may have therapeutic benefit in a subgroup of ALL patients where this locus is intact (148), and demethylating agents are currently in clinical trials for relapsed and refractory ALL. Very little is known about a potential involvement of other epigenetic mechanisms in the biology of Ph^+^ ALL, such as covalent modifications of histones or nucleosome positioning. A better understanding of Ph^+^ ALL biology, including associated genetic and epigenetic abnormalities, should facilitate the development of rational synergistic combinations of targeted agents with TKIs.

### Mechanisms of resistance in Ph^+^ ALL – BCR–ABL1 mutations

**Figure 5:**
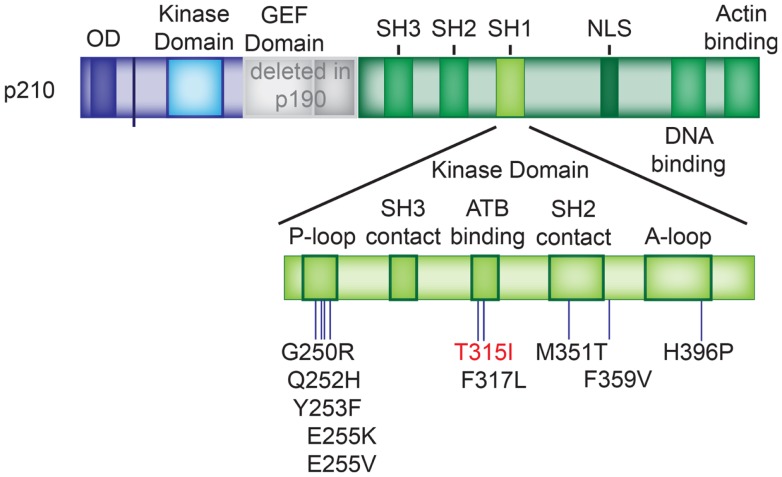
**BCR–ABL1 TKD mutations**. Location of the *BCR–ABL1* tyrosine kinase domain mutations listed in Table 2. The T315I mutation, which causes resistance against imatinib, dasatinib, and nilotinib is depicted in red. P-loop: phosphate-binding loop; A-loop: activation loop.

One of the primary mechanisms of resistance and treatment failure in CML is the acquisition of *BCR*–*ABL1* mutations that render the fusion protein completely or relatively unresponsive to TKIs. A plethora of different mutations mediating imatinib-resistance have been described. Most of these mutant BCR–ABL1 proteins are still sensitive to the second generation ABL kinase inhibitors dasatinib and nilotinib. In addition, the recently approved ponatinib is active against the most common mutation that causes resistance to both first and second generation ABL TKIs, the “gatekeeper” T315I mutation (Table 2; Figure 5). Kinase domain mutations develop even more frequently in Ph^+^ ALL treated with TKI monotherapy despite initial sensitivity (156–162) (161, 163, 164). A rate of BCR–ABL1 kinase domain (TKD) mutations of >80% has been reported in (adult) patients with Ph^+^ ALL at relapse (165–167), with the most common mutations being T315I, Y253H, and E255K/V (42, 168). There has been considerable debate in the field whether these mutations occur during treatment, or whether TKIs select for pre-existing resistant subclones. Several studies suggest that a substantial percentage of patients harbor subclones with TKD mutations prior to the initiation of therapy (42, 169–171). *BCR*–*ABL1* mutations known to cause resistance have been identified in minor subclones in as many as 40% of Ph^+^ patients at initial diagnosis (169). Tyrosine kinase domain mutations may be less common in patients treated with a combination of intensive chemotherapy and TKI. In addition, the kinetics of emergence of resistant mutants – when they do develop – is not well-studied. Sequencing of 10 evaluable pediatric patients treated with imatinib and highly intensive chemotherapy on AALL0031 revealed two known resistance causing BCR–ABL1 mutations at relapse, none of which were detected in the initial diagnostic sample (38). Both mutations are responsive to nilotinib or dasatinib (M244V and H396P) (53). In contrast, in the GIMEMA LAL1205 study, the T315I mutation was discovered in four out of seven patients who relapsed after induction with dasatinib + steroids only, followed by intensive consolidation (intensive chemotherapy + TKI + auto HSCT, or allo HSCT) (172). It is possible that the combination of TKIs with an up-front intensive chemotherapy backbone serves to reduce selective pressure on TKI-resistant subclones. However, more in depth analysis will be required to define the respective roles of resistance to TKI and standard chemotherapy, and their interdependence. The fact that BCR–ABL1 has been found mutated at relapse also raises the important question how aggressively patients with Ph^+^ ALL who receive TKIs on a backbone of standard chemotherapy should be screened for the emergence of TKD mutations. Since patients may still respond to the chemotherapy portion, early warning signs of TKI failure may be missing. If resistant mutants develop or emerge with similar kinetics to what is observed with monotherapy, patients may receive months or years of ineffective TKIs only to ultimately relapse, when early detection of an emergent mutant clone could have prompted switching to another agent active against the mutant BCR–ABL1. A major technical difficulty of such studies is the limit of detection to reliably assess and follow clonal heterogeneity in a minimal residual disease (MRD) setting. The decreased cost of sequencing and novel techniques such as MRD-sort combined with high throughput single cell sequencing may be able to provide answers in the near future. However, the complexity and cost of such an approach would first require a more in depth study of whether BCR–ABL1 mutations are a substantial contributor to resistance and relapse in Ph^+^ ALL treated with intensive chemotherapy plus TKI.

**Table 2:** **Activity (IC50) of imatinib, dasatinib, nilotinib, and ponatinib against selected BCR–ABL1 mutants**.

Domain	Mutant	Imatinib IC50 (nM)	Dasatinib IC50 (nM)	Nilotinib IC50 (nM)	Ponatinib IC50 (nM)
P-loop	G250E	3613	8.14	80.67	*4.1*
	Q252H	733	5.59	46.75	*2.2*
	Y253F	1888	2.89	57.16	*2.8(→H: 6.2)*
	E255K	3174	10. 26	118. 4	*14*
	E255V	8953	6. 30	182. 3	*36*
ATP-binding region	T315I	9221	137. 30	697. 1	*11 (→A: 1.6)*
	F317L	1379	8. 16	39. 19	*1.1 (→V: 10)*
SH2-binding region	M351T	926	1. 61	7. 804	*1.5*
Substrate-binding site	F359V	1509	2. 73	91. 29	*10*
A-loop	H396P	1280	1. 95	42. 65	*1.1*

*IC50 of imatinib, dasatinib, and nilotinib was determined side by side in Ba/F3 cells transfected with the indicated BCR–ABL1 mutants (245) and fairly consistent with previously reported IC50 values in single-agent studies [reviewed in Ref. (246)]. IC50 values for ponatinib were obtained in a separate experiment (247), therefore, direct comparison should be undertaken with caution (*italics*). However, the overall lower IC50 and preserved activity against a wide spectrum of BCR–ABL1 mutations, including T315I, can be appreciated*.

### Mechanisms of resistance in Ph^+^ ALL – increased intracellular BCR–ABL1

In addition to kinase domain mutations, increasing the amount of BCR–ABL1 protein in the cell can lead to resistance to TKIs. One mechanism includes amplification of the *BCR*–*ABL1* fusion gene or the entire Ph^+^ chromosome, which have been reported in up to 10% of Ph^+^ ALL (173, 174). Amplification of p210_BCR–ABL1_ also appears to be particularly common in lymphoid blast crisis of CML (175).

### Mechanisms of resistance in Ph^+^ ALL – overexpression of drug exporters

There is also evidence for the involvement of drug exporters such as ABCB1/MDR (176)1/PGP and ABCG2/BCRP in the development of TKI resistance in CML (112, 176–178). Less data is available on the role of drug efflux pumps in Ph^+^ ALL. One study evaluated the expression levels of LRP, MRP, and ABCB1/MDR1/PGP in Ph^+^ ALL, and found consistent overexpression of LRP, but not MRP and ABCB1/MDR1/PGP (179). In contrast, promoter CpG methylation of MDR1 (i.e., silencing) in ALL was found to be inversely correlated with the presence of the Philadelphia chromosome, suggesting a role for MDR1 in Ph^+^ ALL as well (146). Overexpression of ABCB1/MDR1/PGP was also found as a mechanism of resistance to nilotinib in CML cell lines (180).

### Mechanisms of resistance in Ph^+^ ALL – upregulation of parallel pathways

A large amount of data supports Src-family kinases as a mediator of resistance to imatinib and nilotinib. Src-family kinases are activated downstream of BCR–ABL1, and may play a particularly important role in lymphoid blast crisis and Ph^+^ ALL (see also “Downstream Pathways Activated by BCR–ABL1 Fusion Proteins”) (47, 64–71, 74). Persistent HCK and LYN activity has been detected in CML patients who failed imatinib despite the absence of a BCR–ABL1 mutation (72, 181, 182). In imatinib-resistant cells, LYN was found in a complex with BCR–ABL1 and GAB-2 (72). LYN and HCK have been shown to be activated by BCR–ABL1 in a non-kinase dependent manner, and are in turn able to phosphorylate BCR–ABL (72, 73). Imatinib-resistant CML cell lines or mouse models overexpressing Src-family kinases were shown to respond to the dual BCR–ABL1 – Src-family kinase inhibitor dasatinib, but not nilotinib or Imatinib (147).

Another parallel pathway with a potential role in TKI resistance is the JAK–STAT pathway. p190_BCR–ABL1_ activates JAK1, 2, and 3 and STAT1, 3, 5, and 6, likely providing a positive-feedback loop, substantial signal amplification, and redundancy. A dual BCR–ABL1–JAK2 inhibitor was able to overcome TKI resistance *in vivo* and a K562 mouse model (52, 183). JAK2 inhibitors are currently in clinical trials for myeloproliferative disorders and AML, and are being explored for Ph-like ALL with JAK mutations (184).

An additional escape pathway involves AKT, STAT5, and the B-cell lymphoma 6 (BCL6) transcriptional repressor. BCL6 is a well-established proto-oncogene in lymphoid malignancies that is a common target of activating translocations in diffuse large B-cell lymphoma (DLCBL) (185). STAT5, one of the major downstream targets of the BCR–ABL1 fusion protein, suppresses BCL6 in B-cells (186, 187). *In vitro* treatment of Ph^+^ ALL cells was shown to result in about 90-fold upregulation of BCL6 that was dependent on STAT5, to levels similar to those present in DLCBL. In addition, BCL6 is upregulated by FoxO4, which in turn is inactivated by PI3K/AKT signaling. This suggests involvement of both the JAK2/STAT5 and PI3K/AKT signaling pathways in mediating the derepression of BCL6 in response to TKIs. Deletion, expression of a dominant negative, or pharmacologic inhibition of BCL6 synergized with imatinib and nilotinib in syngeneic and primary patient xenograft mouse models (188). The mechanism of BCL6-mediated resistance may include repression of Arf and p53-mediated apoptosis, and the induction of more stem-cell like features such as quiescence and self-renewal, which may specifically enhance the survival and resistance of LICs (189). Induction of cell cycle exit and quiescence is one of the normal functions of BCL6 during B-cell development at the pre-B stage (190).

The RAS-RAF-MEK–MAPK pathway may also contribute to suboptimal efficacy of BCR–ABL1 inhibitors and resistance (43). MAPK activity was found to be increased in a presumed compensatory response to imatinib treatment of human CD34+ cells transduced with p210_BCR–ABL1_. A MEK-inhibitor reduced proliferation in this model and showed synergy with Imatinib (40). Finally, overexpression of the epidermal growth factor ERBB/HER2/NEU has been linked to drug resistance in ALL (191). ERBB is frequently elevated in Ph^+^ ALL, and ERBB/HER2/NEU inhibitor lapatinib was synergistic with imatinib and nilotinib (but not dasatinib) on Ph^+^ ALL cell lines with high ERBB expression (63).

### Mechanisms of resistance in Ph^+^ ALL – acquisition of additional genetic abnormalities interfering with transcriptional regulation and B-cell development

Mutations of *IKZF1* play a fundamental role in Ph^+^ ALL (86). In addition to being extremely common at diagnosis, *IKZF1* mutations are further enriched at relapse. This includes both presumed *de novo* acquisition of an *IKZF1* mutation (192, 193), as well as selection for a subclone with a more severe mutation (Ik6 or homozygous deletion) at relapse that was initially present at a low percentage (95). This pattern suggests a role for Ikaros in mediating drug resistance and relapse. However, how Ikaros would specifically cause drug resistance is unclear. A potential mechanism could lie in the fact that *IKZF1* mutated B-ALL has a more “stem-cell like” signature by gene-expression profiling than *IKZF1* wild type B-ALL. HSC are highly drug resistant, and expression of a stem-cell program has been associated with drug resistance and poor outcome in other types of leukemia (194–197).

### Mechanisms of resistance in Ph^+^ ALL – inhibition of apoptosis

As discussed above, increased promoter methylation has been described in relapsed ALL specimens when compared to the initial diagnostic specimen. *CDKN2A/B* appears to be a key locus that influences to drug resistance and is affected by epigenetic silencing or deletion (137, 142, 143, 146, 148, 151, 198). One of the consequences of deleting or silencing the *CDKN2A* locus is the inability to upregulate p14^Arf^, which can lead to a loss of functional p53 through increased activity of the p53 E3 ubiquitin ligase HDM2. Another mechanism of inactivating p53 in Ph^+^ ALL involves the overexpression of BCL6 discussed above (188). In a murine model of Ph^+^ ALL, imatinib treatment resulted in BCL6 upregulation and downregulation of p53, while genetic inactivation of BCL6 resulted in increased levels of p53 and failure to cause leukemia in mice. Interestingly, BCL6 was also recruited to the *CDKN2A* locus. Dual inhibition of BCR–ABL1 and BCL6 demonstrated in synergy in xenografts of patient-derived primary Ph^+^ ALL cells. Efficient inactivation of the p53 pathway by BCR–ABL1 and its downstream targets may explain why genetic mutations or deletions of p53 are rare up-front in Ph^+^ ALL (199, 200). Acquisition of p53 mutations at relapse has been described, but appears a rather rare event compared to the commonly affected *IKZF1, PAX5*, and *CDKN2A/B* loci (150, 201–203).

### Mechanisms of resistance in Ph^+^ ALL – resistance to concomitant chemotherapeutic agents

In addition to resistance to TKIs, resistance to standard chemotherapy agents can develop during treatment with chemotherapy plus TKI. Mechanisms of resistance discussed above – mutation, amplification, or upregulation of the molecular target, drug exporters, the expression of stem-cell signatures, upregulation of survival pathways, and inhibition of apoptosis apply to cytotoxic agents as well (204–206). In addition, several recurrent genetic alterations have been found enriched in ALL at relapse that confer resistance to specific cytotoxic agents. These include loss of *MSH6* [mediating resistance to thiopurines, alkylating agents, and prednisone (202)], decreased MSH2 protein levels (mediating resistance to purine analogs) (207), and mutations in *NR3C1* and *CREBBP* (mediating glucocorticoid resistance) (55, 193, 203, 208–211). None of these mechanisms appear to be specific to Ph^+^ ALL. Finally, the contribution of AKT and MTOR – both are activated in response to BCR–ABL1 signaling – to glucocorticoid resistance has been discussed above (55, 56).

### Mechanisms of resistance in Ph^+^ ALL – interactions with the bone marrow niche and the immune system

In addition to cell-autonomous mechanisms of resistance, ALL blasts interact with the bone marrow niche (212) and the immune system. Some of these interactions probably contribute to the emergence of resistance and relapse. At the same time, our improved understanding of leukemic blast–host interactions has led to the development of therapeutic approaches that attempt to disrupt the interaction with the bone marrow niche (213, 214), or aim to break tolerance and effectively engage the patient’s immune system in the eradication of leukemia [reviewed in Ref. (206)]. None of these approaches are specifically targeted at Ph^+^ ALL.

## Current Clinical Concepts in the Treatment of Ph^+^ ALL

### Initial studies using imatinib

Prior to the use of TKI, survival for pediatric Ph^+^ ALL treated with chemotherapy with or without HSCT was extremely poor. The International Ponte di Legno Childhood ALL Consortium reported 7-year event-free survival (EFS) and OS rates of 25 and 36% for 326 Ph^+^ ALL patients diagnosed between 1985 and 1996, and 32 and 45% for 610 Ph^+^ patients diagnosed between 1995 and 2005 and treated without TKI in first remission (4, 215). Although initial white blood cell count, age, and early response to therapy were predictive of outcome, the EFS even for “good-risk” patients was <50% (4). In the pre-TKI era, HSCT in first remission was generally considered to be the best available treatment option, particularly if a matched related donor was available. While HSCT produced slightly better results than chemotherapy alone, relapse was common even after allogeneic HSCT. In the 1995–2005 era, the 5-year EFS and OS rates for patients who achieved a first remission (89%) and went on to HSCT were only 34.2 and 48.3% (4).

**Figure 6:**
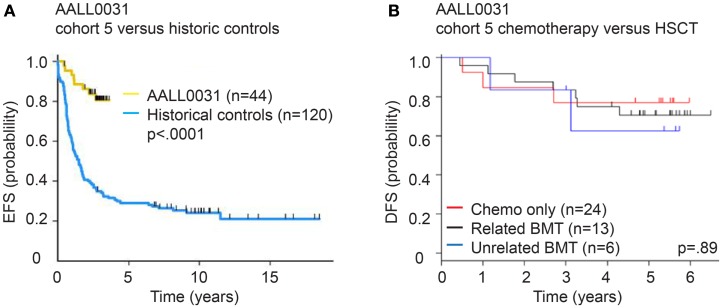
**Survival of children with Ph^+^ ALL treated with imatinib + chemotherapy on COG AALL0031**. **(A)** Event-free survival (early follow-up) of cohort 5 of AALL0031 treated with the MTD of imatinib in combination with chemotherapy compared to historic controls. Thirteen of the 44 patients went on to receive a matched related HSCT, while the remaining 31 patients received chemotherapy only. Introduction of imatinib onto a backbone of standard chemotherapy dramatically improved the outcome of Ph^+^ ALL. **(B)** Disease-free survival (5.2-year median follow-up) of patients treated as per AALL0031 cohort 5 based on transplant status. Chemotherapy only – 24 patients, matched related HSCT on study – 13 patients, unrelated HSCT off study – 6 patients. HSCT did not offer additional benefit compared to chemotherapy + imatinib.

Following the landmark reports showing the efficacy of imatinib in CML, several studies had shown transient single-agent efficacy of imatinib in patients with relapsed or refractory Ph^+^ ALL, and adult trials had shown the feasibility of combining imatinib with chemotherapy, albeit on an intermittent dosing schedule and typically as a bridge to early HSCT (157, 159, 162). In 2002, the Children’s Oncology Group (COG) began the AALL0031 trial that was designed to evaluate the safety and efficacy of combining imatinib (340 mg/m^2^/day) with a very intensive chemotherapy regimen (2). Imatinib was introduced after 4-weeks of induction therapy were completed and the exposure was successively increased in five cohorts, with expanded accrual in cohort 5 to estimate efficacy. In cohort 5, patients received continuous imatinib therapy starting at day 1 of consolidation therapy, with intermittent (14 days on – 14 days off) dosing during the last year of maintenance therapy. A parallel cohort of Ph^−^ very high-risk ALL patients was treated with the identical chemotherapy regimen, without imatinib. Overall, addition of imatinib to the intensive chemotherapy regimen was tolerated very well, with no significant increase in toxicity of the combined regimen compared to chemotherapy alone. In the initial study report, the 3-year EFS for patients treated in cohort 5 was 80%, as compared to 35% in a historical control group treated in the pre-TKI era (Figure 6) (2). This outstanding outcome has been stable over time with recent analyses showing a 71% 7-year EFS rate for patients in AALL0031 cohort 5 versus 21.4% in the historical control group (Figure 6) (216). AALL0031 also explored the use of HSCT (matched related HSCT for patients on study, the final analysis also included follow-up of patients who went off study for an unrelated-donor HSCT). Neither matched related or unrelated HSCT offered any additional benefit (2, 216). Results of this trial, although based on a relatively small number of patients, revolutionized clinical practice for children and adolescents with Ph^+^ ALL. Tyrosine kinase inhibition is an integral part of treatment for Ph^+^ ALL and is now incorporated during induction therapy. HSCT in first remission is no longer routinely recommended for all patients as a default, though it may still be an important treatment for patients that respond poorly to chemotherapy plus TKI.

A key pediatric study conducted primarily in Europe by 10 study groups in parallel to COG AALL0031, was the EsPhALL phase 3 trial (91). Patients received induction therapy according to the standard of their respective study group, and were categorized as good (108 patients) or poor risk (70 patients) based on initial response. The study then randomized good-risk patients to chemotherapy with or without post-induction imatinib on the backbone of the intense Associazione Italiana di Ematologia Oncologica Pediatrica – Berlin–Frankfurt–Münster (AIEOP-BFM) ALL 2000 regimen. Poor-risk patients were all assigned to chemotherapy plus imatinib. HSCT was recommended for all patients, and performed in 77% of patients in this trial. The imatinib exposure was intermittent with much lower cumulative exposure than used on AALL0031 (126 versus 616 days on AALL0031 cohort 5). The EsPhALL trial confirmed the superior efficacy of imatinib plus chemotherapy. The 4-year disease-free survival (as treated analysis of 81 patients) was 75.2% for good-risk patients receiving imatinib and 55.9% for those who did not receive imatinib (*p* = 0.06). Outcomes for the poor-risk population were also encouraging with 4-year EFS of 53.5%, which was significantly better than the outcome of historical control patients treated without imatinib. This is the largest, and the only randomized trial to systematically assess imatinib in combination with intensive multi-agent chemotherapy in children with Ph^+^ ALL. Given the results of this trial and AALL0031, it is highly unlikely that another trial will ever be conducted for chemotherapy with/without TKI in pediatric Ph^+^ ALL. The EsPhALL trial was amended to start continuous imatinib treatment at day 15 of induction therapy, and decrease the number of patients who underwent HSCT in first remission. This study will accrue patients through early to mid 2014.

A superior outcome with the addition of imatinib to chemotherapy in pediatric Ph^+^ ALL was also reported by the Sociedad Española de Hematología y Oncología Pediátricas (217). In the SHOP-2005 trial imatinib was initiated on day 15 of induction, and most patients (15/16) underwent bone marrow transplantation in first remission. The 3-year EFS for patients on this trial was significantly higher than that of historical controls who did not receive imatinib (the rate of BMT in the historical control was 17/27).

### Second generation TKIs (dasatinib, nilotinib)

In light of the encouraging but still suboptimal results obtained with chemotherapy plus imatinib in pediatric Ph^+^ ALL, and the development of second generation BCR–ABL1 inhibitors, it was logical to investigate whether the use of dasatinib or nilotinib in combination with chemotherapy might further improve outcomes (Table 3). Multiple lines of reasoning suggest that this could be the case. Second generation TKIs are more potent inhibitors of BCR–ABL1 *in vitro*, and lead to earlier and more profound reduction of the leukemic clone burden in CML (15, 16). The CNS is a well-known sanctuary site in ALL and CNS penetration of imatinib is poor. While very little data are available on the CNS penetration of nilotinib, oral administration of dasatinib produces therapeutic levels in the CSF (218). The imatinib-resistant TKD mutations reported to occur in relapsed Ph^+^ ALL are typically responsive to dasatinib or nilotinib, with the important exception of the gatekeeper T315I mutation that is resistant to all three agents (38, 53). Finally, multiple other kinases are activated by BCR–ABL1, including PI3K/AKT (39, 40), EGFR, MAP-kinase (40, 45), JNK/SAPK, JAK1–3, and the Src-family kinases LYN, HCK, and FGR (47, 67–71). Particularly Src-kinases appears to be an important downstream target of the BCR–ABL1-induced signaling cascade, while also phosphorylating BCR–ABL1 in a positive-feedback loop, and contributing to clinical resistance against TKIs. This pathway may be particularly important in Ph^+^ lymphoid malignancies (47, 72, 74, 91, 157, 180–182). Because dasatinib targets both BCR–ABL1 and Scr-family kinases, it is a particularly attractive agent to investigate in Ph^+^ ALL, as it may suppress the BCR–ABL1 signaling cascade at multiple levels. This could result in a more profound inhibition, reduction of the emergence of resistance, and improved clinical outcomes. In contrast to dasatinib, nilotinib does not efficienty inhibit Src-family kinases, and LYN (as well as two other tyrosine kinases that interact with LYN, SYK and AXL) has been implicated in mediating resistance to nilotinib (180, 219).

**Table 3:** **Selected open clinical trials (clinicaltrials.gov) investigating dasatinib and nilotinib for Ph^+^ ALL**.

Identifier	Title	Phase	Study group	Age (years)	Backbone	HSCT	TKI duration
NCT01460160 (dasatinib)	Pediatric Ph^+^ ALL (CA180–372)	II	Multi-center	1–18	AIEOP-BFM 2000	Based on MRD	2 years
NCT01256398 (dasatinib)	Dasatinib followed by HSCT for Ph^+^ all	II	CALGB, ECOG SWOG	>50	Dex, VCR, 6MP Dauno, VP-16, MTX, CXP	All pt	Indefinite
NCT01724879 (dasatinib)	Frontline dasatinib plus chemotherapy in Ph^+^ ALL	III	GMALL	18–55	GMALL 07/2003	All pt	Indefinite
NCT00792948 (dasatinib)	Hyper-CVAD + dasatinib with or without HSCT for Ph^+^ ALL	II	NCI	18–60	Hyper-CVAD	All Pt	5 years
NCT01077544 (nilotinib)	A PK study of nilotinib in pediatric Ph^+^ ALL	I	Multi-center	<18	Monotherapy for relapsed/refractory ALL	N/A	N/A
NCT01670084 (nilotinib)	Nilotinib and combination chemotherapy in newly diagnosed Ph^+^ ALL	II	Mayo Clinic, USA	18–70	Hyper-CVAD ± nilotinib ± rituximab	No	Through maint.
NCT01528085 (nilotinib)	Nilotinib in combination with chemotherapy in elderly Ph^+^ ALL patients	II	Goethe University, Germany	>55	Dex, VCR, 6MP, MTX, CXP, AraC	No	Through maint.
NCT00844298 (nilotinib)	Nilotinib and combination chemotherapy in newly diagnosed Ph^+^ ALL	II	Asan Medical Center	>15	Dauno, VCR, AraC, Pred, MTX	All Pt	2 years in non-HSCT patients
NCT01914484 (nilotinib)	Nilotinib/ruxolitinib for TKI-resistant Ph^+^ leukemia	I/II	University Health Network	>18	None	N/A	N/A

*AIEOP, Associazione Italiana di Ematologia Pediatrica (Italy); BFM, Berlin–Frankfurt–Münster (Germany); CALGB, Cancer and Leukemia Group B (USA); ECOG, Eastern Cooperative Oncology Group (USA); SWOG, Southwest Oncology Group (USA); GMALL, German Multicenter ALL Working Group (Germany); NCI, National Cancer Institute (USA), Asan Medical Center, Seoul, Korea; University Health Network, Toronto, ON, Canada; MRD, minimal residual disease*.

Dasatinib monotherapy demonstrated encouraging efficacy against adult Ph^+^ ALL in early clinical trials (220, 221). In the GIMEMA LAL1205 study, 55 patients received induction with dasatinib + steroids and IT-MTX, while post-induction consolidation was at the discretion of the treating center. Two patients received no further therapy and 19 patients continued on TKI only (69.6% relapse at 20 months), 14 patients received intensive chemotherapy + TKI (21.7% relapses), 18 went on to allogeneic HSCT (11.1% relapses) (172). This trial demonstrated impressive remission rates for dasatinib and steroids only, but at the same time underscored the importance of adding intensive chemotherapy and/or HSCT to maintain durable remissions. The encouraging early data led to several trials investigating dasatinib in combination with chemotherapy. Incorporation of pulses of twice daily dasatinib with hyper-CVAD, followed by continuous dasatinib during maintenance and indefinitely after completion of chemotherapy resulted in a CR rate of 94% and an estimated 2-year survival of 64% (EFS and OS have not yet been reported) (222). The combination of dasatinib with hyper-CVAD using a similar regimen (but with dasatinib given continuous) in adults with relapsed Ph^+^ ALL was also recently reported (223). Dasatinib needed to be dose reduced from 100 to 70 mg daily due to prolonged cytopenias. The lower dose was well-tolerated and the CR rate was encouraging at 68%, but only two patients (11%) were alive with at a median follow-up of 52 months.

Two phase I/II pediatric trials showed that dasatinib monotherapy was well-tolerated and safe in children (224, 225). The COG AALL0622 trial tested dasatinib (60 mg/m^2^/day) in combination with the same chemotherapy regimen used with imatinib in COG AALL0031. In addition to the different TKIs used in the two studies, AALL0622 started dasatinib at day 15 of induction rather than starting after induction therapy was completed as done in AALL0031. AALL0622 has completed accrual and concluded that daily dasatinib therapy was safe with this chemotherapy regimen (226). Although it is too early to report outcome following treatment with chemotherapy plus dasatinib on AALL0622, the early response rates compare favorably to those obtained in AALL0031 with chemotherapy plus imatinib (227). The complete remission and end block 2 consolidation MRD negative rates were 98 and 89% on AALL0622, as compared to 89 and 71% on AALL0031. The COG and the European EsPhALL group are currently collaborating on a study that tests adding dasatinib 60 mg/m^2^/day to the EsPhALL chemotherapy backbone (COG AALL1122, NCT01460160) and are working together to develop a successor trial that randomizes patients to receive one of two different chemotherapy backbones plus TKI.

The other major second generation TKI is nilotinib, which has similar activity to dasatinib against imatinib-resistant BCR–ABL1 mutants. However, nilotinib does not inhibit the Src-family kinases, which is theoretically important in Ph^+^ ALL but has not yet been shown to be clinically important (180, 219). It is not clear whether the CNS penetration of nilotinib is as good as that of dasatinib (218). Nevertheless, nilotinib has shown encouraging activity in adult relapsed/refractory Ph^+^ ALL, with some patients achieving responses that were sustained for months on monotherapy (228, 229). Published studies on nilotinib therapy in newly diagnosed Ph^+^ ALL are scarce, but a recently presented pilot study of four Ph^+^ ALL patients who received a combination of nilotinib with intensive chemotherapy reported complete remissions in all patients (230). Early results of combining nilotinib with intensive chemotherapy in the current South Korean cooperative trial have also shown excellent activity (231). Nilotinib was given continuously from induction until completion of chemotherapy (2 years). All patients were eligible for HSCT, and 59/91 of patients underwent HSCT in first remission. The complete remission rate was 90% and the 2-year OS 70%.

### Targeting the T315I mutation: Ponatinib

Dasatinib and nilotinib are effective against the vast majority of reported BCR–ABL1 TKD mutants with one crucial exception: the T315I mutation. For many years, T315I conferred resistance to all available TKIs. Ponatinib is a multi-targeted TKI with strong activity against BCR–ABL and imatinib-resistant mutants including, for the first time, T315I. Ponatinib gained accelerated approval from the FDA in the US in December 2012 based on very promising activity observed in the PACE II clinical trial, including major cytogenetic responses in 70% of patients with T315I mutations (18). Several other studies have shown promising activity in newly diagnosed CML patients (NCT01570868, NCT01641107, and the EPIC trial NCT01650805), but all of these US trials were halted in October 2013, when a high rate of cardiovascular adverse events was reported in post-approval surveillance. Ponatinib was approved for use in the European Union in July 2013, and remains on the market at this time. As of December 2013, the future of this agent, which is the only agent known to be effective for CML and Ph^+^ ALL patients with T315I mutations, remains uncertain. Concerningly high rates of cardiovascular events were also reported for nilotinib, although they have received less attention in the scientific and lay press (232, 233). This raises the important question how to weigh molecular efficacy against risks and side effects of treatment. The risk–benefit ratio of any given agent will be different in an elderly patient with cardiovascular risk factors or pre-existing disease with newly diagnosed CML, or a child with Ph^+^ ALL. Irrespective of its potential use in TKI-naïve CML and Ph^+^ ALL patients, the impressive activity against the T315I BCR–ABL1 will likely make ponatinib a crucial second line agent for patients with this mutation.

## Current and Future Clinical Questions

### Chemotherapy backbone

In addition to the choice of TKI to be used in Ph^+^ ALL, several other critical questions regarding the optimal therapy for pediatric Ph^+^ ALL remain unresolved. The current highly intense chemotherapy backbones are associated with substantial treatment related morbidity and mortality, as well as significant potential risk of late effects such as infertility and second malignancies. Furthermore, chemotherapy that is too intensive may compromise the ability to deliver optimal TKI therapy or to combine TKIs with other new targeted therapies. As discussed above, COG AALL1122, NCT01460160 is investigating dasatinib on the chemotherapy backbone of the AEIOP-BFM ALL 2000 regimen, which had shown excellent results in the EsPhALL trial. This backbone contains significantly reduced cumulative doses of cyclophosphamide, ifosfamide, etoposide, and high-dose methotrexate compared to AALL0031. Despite the changes, the morbidity and mortality associated with this regimen is still substantial. A successor trial in development proposes to compare the EsPhALL backbone to a less intensive chemotherapy regimen, with identical TKI therapy in the two regimens.

### Duration of TKI inhibition

Another open question is the duration of TKI inhibition. Several current protocols in adult patients propose indefinite tyrosine kinase inhibition after completion of maintenance ALL therapy (Table 1). In contrast, the current pediatric Ph^+^ ALL trials typically stop TKI therapy when chemotherapy stops at 2–2.5 years post diagnosis. There have been some, but relatively few late events in COG AALL0031 suggesting that this approach is feasible and not associated with excessive relapses. However, it remains uncertain if additional TKI therapy beyond 2–2.5 years is beneficial. In particular, there are concerns about growth and bone mineral density with prolonged TKI therapy in children (234–236). These effects may be compounded by the concomitant use of highly intensive chemotherapy and radiation, both of which have been shown to independently affect longitudinal growth and bone health (237, 238).

### The role of hematopoietic stem-cell transplant

In AALL0031, HSCT did not improve outcomes for patients treated with imatinib in the final cohort. In contrast, outcomes for patients who did not undergo HSCT on EsPhALL appeared inferior to the transplanted cohort (relapse was reported in three of nine good-risk patients and five of seven poor-risk patients). It is possible that the longer duration of imatinib on AALL0031 (616 versus 126 days on EsPhALL) plays a role. On both trials, the numbers were extremely small, and more data will be required to define the role of HSCT in Ph^+^ ALL. The current combined follow-up study of AALL0031/AALL0622 and EsPhALL, AALL1122 (NCT01460160), is assessing the effect of earlier, continuous, and longer exposure to dasatinib. Rather than pursuing HSCT in all patients, only patients who fail to meet predefined MRD criteria and have a suitable donor are assigned to undergo HSCT in first remission, which is the approach generally pursued in other pediatric ALL subsets. In adult patients, initial studies were unable to address the question of whether HSCT confers a survival benefit due to small sample sizes, but durable remissions have been observed with the combination of TKI and intensive chemotherapy regimens (157, 159, 162). HSCT in first remission (if possible based on donor availability and clinical status of the recipient) remains the standard of care in younger adults with Ph^+^ ALL, but outstanding results observed with chemotherapy plus TKI are challenging this consensus. Despite controversy over the role of HSCT in first remission in pediatric Ph^+^ ALL, there is universal consensus that HSCT in second complete remission is the preferred therapy after relapse.

### The role of post-transplant TKI

Another open question centers on whether and for how long TKIs could or should be continued after HSCT. Several studies suggest that post-transplant TKI are beneficial and reduce the rate of relapse (239–242). However, the duration of post-transplant TKI therapy varies widely between studies, and high rates of dose reduction and cessation due to cytopenias and other side effects have been reported. Very few prospective randomized studies are available. A recent GMALL study randomly assigned Ph^+^ ALL patient to receive imatinib post-HSCT either prophylactically, or triggered by a rise in MRD. Molecular recurrence after HSCT was significantly lower in the prophylactic group (40 versus 69%; *P* = 0.046), although this did not translate into a difference in survival (243). In a recently reported non-randomized trial of 92 patients with Ph^+^ ALL, the ability to tolerate post-HSCT imatinib was an independent predictor of DFS and OS (244). This study involved pediatric patients, who had a higher rate of tolerating post-transplant imatinib than adult patients. In the current pediatric AALL1122, NCT01460160 post-transplant imatinib for up to 12 additional months is optional and at the discretion of the treating investigator.

## Conclusion

Ph^+^ ALL is a poster child for the successful integration of targeted small-molecule inhibitors with standard chemotherapy. TKIs have revolutionized the outcomes of this disease. However, survival rates are still inferior to most other types of childhood ALL. Future efforts to improve survival and decrease toxicity need to focus on refining chemotherapy regimens, and optimizing TKI therapy. In addition, increasing the mechanistic understanding of this complex and fascinating disease will facilitate translating new findings into improved targeted therapies.

## Conflict of Interest Statement

The authors declare that the research was conducted in the absence of any commercial or financial relationships that could be construed as a potential conflict of interest.

## Abbreviations

ALL, acute lymphoblastic leukemia; CML, chronic myeloid leukemia; DFS, disease-free survival; EFS, event-free survival; HSCT, hematopoietic stem-cell transplantation; OS, overall survival; Ph^+^, Philadelphia chromosome positive; TKD, tyrosine kinase domain; TKI, tyrosine kinase inhibitor.
